# Supplementary material for: Antioxidative effects of molybdenum and its association with reduced prevalence of hyperuricemia in the adult population
Source: PLoS One. 2024 Aug 1;19(8):e0306025. doi: 10.1371/journal.pone.0306025 (PMC11293656; doi:10.1371/journal.pone.0306025)
Supplement: S1 Table — (DOCX) [file pone.0306025.s001.docx]

**S1 Table.** Urinary molybdenum levels in relation to eGFR

| eGFR, mL/min/1.73 m^2^  (N = 15,370) | N | Urinary molybdenum-to-creatinine ratio (ng/mg) | *P* value |
| --- | --- | --- | --- |
| eGFR categories |  |  | 0.008 |
| >90 | 8,936 | 50.5 ± 41.2 |  |
| 60−90 | 5,007 | 48.9 ± 42.5 |  |
| 30−60 | 1,291 | 48.1 ± 36.1 |  |
| 15−30 | 107 | 47.4 ± 31.0 |  |
| <15 | 29 | 30.4 ± 19.6 |  |

Abbreviation: eGFR, estimated glomerular filtration rate
