# Supplementary material for: Antioxidative effects of molybdenum and its association with reduced prevalence of hyperuricemia in the adult population
Source: PLoS One. 2024 Aug 1;19(8):e0306025. doi: 10.1371/journal.pone.0306025 (PMC11293656; doi:10.1371/journal.pone.0306025)
Supplement: S2 Table — (DOCX) [file pone.0306025.s002.docx]

**S2 Table.** Sensitivity analysis for the association between urinary molybdenum and prevalence of hyperuricemia

|  | Model 1 |  | Model 2 |  | Model 3 |  |
| --- | --- | --- | --- | --- | --- | --- |
|  | OR (95% CI) | *P* value |  | *P* value | OR (95% CI) | *P* value |
| Urinary molybdenum-to-creatinine ratio |  | < 0.001 |  | < 0.001 |  | < 0.001 |
|  |  | < 0.001^*^ |  | < 0.001^*^ |  | < 0.001^*^ |
| Q1, reference | 1 |  | 1 |  | 1 |  |
| Q2 | 0.87 (0.77–0.98) | 0.022 | 0.87 (0.77–0.98) | 0.027 | 0.84 (0.74–0.96) | 0.009 |
| Q3 | 0.78 (0.69–0.88) | < 0.001 | 0.79 (0.70–0.90) | < 0.001 | 0.78 (0.68–0.89) | < 0.001 |
| Q4 | 0.73 (0.64–0.83) | < 0.001 | 0.74 (0.65–0.84) | < 0.001 | 0.70 (0.61–0.80) | < 0.001 |

Abbreviation: Q1−Q4, quartile group of urinary molybdenum levels.

^*^*P*-for-trend

Hyperuricemia is defined as a serum uric acid concentration of over 6.0 mg/dL for females and over 7.0 mg/dL for males.

Multivariable logistic regression analysis of model 1 was adjusted for age, sex, ethnicity, BMI, diabetes mellitus, hypertension, and estimated glomerular filtration rate. Model 2 included covariates of model 1 and survey cycle, serum albumin levels. Model 3 included covariates of model 2 and socioeconomic status, education levels, and smoking.
