# Supplementary material for: Antioxidative effects of molybdenum and its association with reduced prevalence of hyperuricemia in the adult population
Source: PLoS One. 2024 Aug 1;19(8):e0306025. doi: 10.1371/journal.pone.0306025 (PMC11293656; doi:10.1371/journal.pone.0306025)
Supplement: S3 Table — (DOCX) [file pone.0306025.s003.docx]

**S3 Table.** Subgroup analysis for the association between urinary molybdenum and prevalence of hyperuricemia according to sex.

|  | Male group (N = 7,566) |  | Female group (N = 7,804) |  |
| --- | --- | --- | --- | --- |
|  | OR (95% CI) | *P* value |  | *P* value |
| Urinary molybdenum-to-creatinine ratio |  | < 0.001 |  | 0.039 |
|  |  | < 0.001^*^ |  | 0.004^*^ |
| Q1, reference | 1 |  | 1 |  |
| Q2 | 0.83 (0.71–0.97) | 0.016 | 0.91 (0.75–1.10) | 0.326 |
| Q3 | 0.73 (0.62–0.86) | < 0.001 | 0.83 (0.68–1.01) | 0.067 |
| Q4 | 0.70 (0.58–0.83) | < 0.001 | 0.75 (0.61–0.92) | 0.005 |

Abbreviation: Q1−Q4, quartile group of urinary metal levels.

^*^*P*-for-trend

Hyperuricemia is defined as a serum uric acid concentration of over 6.0 mg/dL for females and over 7.0 mg/dL for males.

Multivariable logistic regression analysis of model 1 was adjusted for age, sex, ethnicity, BMI, diabetes mellitus, hypertension, and estimated glomerular filtration rate.
