# Supplementary material for: Antioxidative effects of molybdenum and its association with reduced prevalence of hyperuricemia in the adult population
Source: PLoS One. 2024 Aug 1;19(8):e0306025. doi: 10.1371/journal.pone.0306025 (PMC11293656; doi:10.1371/journal.pone.0306025)
Supplement: S4 Table — (DOCX) [file pone.0306025.s004.docx]

**S4 Table.** Subgroup analysis for the association between urinary molybdenum and prevalence of hyperuricemia according to age

|  | 18 ≤ Age < 30  (N = 3,569) |  | 30 ≤ Age < 45  (N = 3,725) |  | 45 ≤ Age < 65  (N = 4,611) |  | Age ≥ 65  (N = 3,465) |  |
| --- | --- | --- | --- | --- | --- | --- | --- | --- |
|  | OR (95% CI) | *P* value | OR (95% CI) | *P* value | OR (95% CI) | *P* value | OR (95% CI) | *P* value |
| Urinary molybdenum-to-creatinine ratio |  | 0.006 |  | 0.004 |  | < 0.001 |  | 0.532 |
|  |  | < 0.001^*^ |  | < 0.001^*^ |  | < 0.001^*^ |  | 0.368^*^ |
| Q1, reference | 1 |  | 1 |  | 1 |  | 1 |  |
| Q2 | 0.79 (0.60–1.05) | 0.103 | 0.76 (0.59–0.96) | 0.038 | 0.80 (0.65–0.99) | 0.038 | 1.09 (0.85–1.40) | 0.516 |
| Q3 | 0.69 (0.51–0.92) | 0.013 | 0.66 (0.50–0.86) | 0.002 | 0.75 (0.61–0.94) | 0.012 | 0.92 (0.71–1.18) | 0.511 |
| Q4 | 0.56 (0.40–0.79) | 0.001 | 0.63 (0.46–0.86) | 0.003 | 0.63 (0.50–0.80) | < 0.001 | 0.94 (0.74–1.20) | 0.634 |

Abbreviation: Q1−Q4, quartile group of urinary metal levels.

^*^*P*-for-trend

Hyperuricemia is defined as a serum uric acid concentration of over 6.0 mg/dL for females and over 7.0 mg/dL for males.

Multivariable logistic regression analysis of model 1 was adjusted for age, sex, ethnicity, BMI, diabetes mellitus, hypertension, and estimated glomerular filtration rate.
