# Supplementary material for: Antioxidative effects of molybdenum and its association with reduced prevalence of hyperuricemia in the adult population
Source: PLoS One. 2024 Aug 1;19(8):e0306025. doi: 10.1371/journal.pone.0306025 (PMC11293656; doi:10.1371/journal.pone.0306025)
Supplement: S5 Table — (DOCX) [file pone.0306025.s005.docx]

**S5 Table.** Subgroup analysis for the association between urinary molybdenum and prevalence of hyperuricemia according to body mass index.

|  | BMI < 25  (N = 4,846) |  | 25 ≤ BMI < 30  (N = 5,108) |  | BMI ≥ 30  (N = 5,188) |  |
| --- | --- | --- | --- | --- | --- | --- |
|  | OR (95% CI) | *P* value | OR (95% CI) | *P* value | OR (95% CI) | *P* value |
| Urinary molybdenum-to-creatinine ratio |  | 0.609 |  | < 0.001 |  | 0.003 |
|  |  | 0.813 |  | < 0.001^*^ |  | < 0.001^*^ |
| Q1, reference | 1 |  | 1 |  | 1 |  |
| Q2 | 0.82 (0.61–1.10) | 0.181 | 0.80 (0.65–0.99) | 0.038 | 0.94 (0.79–1.11) | 0.476 |
| Q3 | 0.93 (0.69–1.25) | 0.631 | 0.71 (0.57–0.89) | 0.002 | 0.78 (0.65–0.93) | 0.006 |
| Q4 | 0.93 (0.69–1.25) | 0.623 | 0.63 (0.51–0.78) | < 0.001 | 0.73 (0.60–0.89) | 0.002 |

Abbreviation: BMI, body mass index, Q1−Q4, quartile group of urinary metal levels.

^*^*P*-for-trend

Hyperuricemia is defined as a serum uric acid concentration of over 6.0 mg/dL for females and over 7.0 mg/dL for males.

Multivariable logistic regression analysis of model 1 was adjusted for age, sex, ethnicity, BMI, diabetes mellitus, hypertension, and estimated glomerular filtration rate.
