# Supplementary material for: Antioxidative effects of molybdenum and its association with reduced prevalence of hyperuricemia in the adult population
Source: PLoS One. 2024 Aug 1;19(8):e0306025. doi: 10.1371/journal.pone.0306025 (PMC11293656; doi:10.1371/journal.pone.0306025)
Supplement: S1 Method — (DOCX) [file pone.0306025.s006.docx]

**S1 Method.** Culture of HK-2 cells, cell viability assay, western blot analysis, and fluorescent microplate assay

Culture of HK-2 cells

HK-2 human kidney proximal tubule epithelial cell line (Korean Cell Line Bank, Korea) was cultured in Dulbecco's modified Eagle medium-F12 mixture (Biowest, France) supplemented with 10% fetal bovine serum and penicillin-streptomycin at 37°C and 5% CO_2_. Cells were seeded onto 6-well plates at 2×10^5^ cells per well, initially in complete culture media for 24 h, then starved for 24 h in serum-free media before the experimental treatment.

Cell viability assay

To determine the effect of molybdenum on cell survival and proliferation, a thiazolyl blue tetrazolium bromide (MTT; Sigma) assay was performed. HK-2 cells were incubated in 96-well plates (8×10^3^/well) in complete culture media for 24 h, then in serum-free media for 24 h. Following serum starvation, cells were treated up to 400 μg/mL of molybdenum for 24 h. Following MTT (0.5 mg/mL dH_2_O) incubation for 4 h at 37°C, dimethyl sulfoxide was added to dissolve the resultant formazan. The absorbance of the resulting solution was measured at 570 nm using a microplate reader (spectraMax Plus 384; Molecular Devices, USA).

Western blot analysis

To observe cellular oxidative stress and antioxidative defense at the protein level, western blotting was performed. Cells were washed with phosphate-buffered saline (PBS) and then harvested in radioimmunoprecipitation assay buffer following 24-h exposure to molybdenum and H_2_O_2_. Proteins extracted from cells were quantified through bicinchoninic acid assay and prepared for electrophoresis by mixing equal amounts with 5× sodium dodecyl sulfate-polyacrylamide gel electrophoresis (SDS-PAGE) loading dye and boiling for 10 min. Samples were separated on 12% SDS-PAGE gels and transferred to polyvinylidene fluoride membranes (Sigma). Membranes were blocked for a minimum of 1 h following transfer and incubated overnight at 4°C with primary antibodies for manganese superoxide dismutase (MnSOD) (1:500; Cell Signaling Technology, USA) or β-actin (1:10 000; Sigma). Membranes were then washed thrice in Tris-buffered saline with Tween 20 and incubated in a blocking buffer solution with the appropriate mouse or rabbit secondary antibody (Cell Signaling Technology). Following secondary antibody incubation, membranes were washed again in Tween 20 thrice. Each membrane was then developed using the WesternBright ECL kit (Advansta, USA) via the ImageQuant LAS4000 system and each band was quantified relative to control.

Fluorescent microplate assay

Intracellular ROS was evaluated by a fluorescent microplate assay using the 2',7'–dichlorofluorescein diacetate (DCFDA) Cellular ROS Detection Assay Kit (Abcam, USA). HK-2 cells were seeded in 96-well plates at 8×10^3^/well in complete culture medium for 24 h, then in serum-free medium for the subsequent 24 h. Then according to the manufacturer’s protocols, the cells were washed in 1× Buffer, treated with 25 μM DCFDA solution for 45 mins, then washed in 1× Buffer again. After washing, cells were treated with molybdenum and 200 μM H_2_O_2_ for 2 h. Immediately following 2 h-incubation, fluorescence was measured using the VICTOR multilabel plate reader (Perkin-Elmer, USA) at excitation/emission spectra of 485/535 nm.
