# Supplementary material for: Antioxidative effects of molybdenum and its association with reduced prevalence of hyperuricemia in the adult population
Source: PLoS One. 2024 Aug 1;19(8):e0306025. doi: 10.1371/journal.pone.0306025 (PMC11293656; doi:10.1371/journal.pone.0306025)

Supplementary Material (Original Western Blot)

Antioxidative effects of molybdenum and its association with reduced prevalence of hyperuricemia in the adult population

1. 2019 08 29 Western Blot

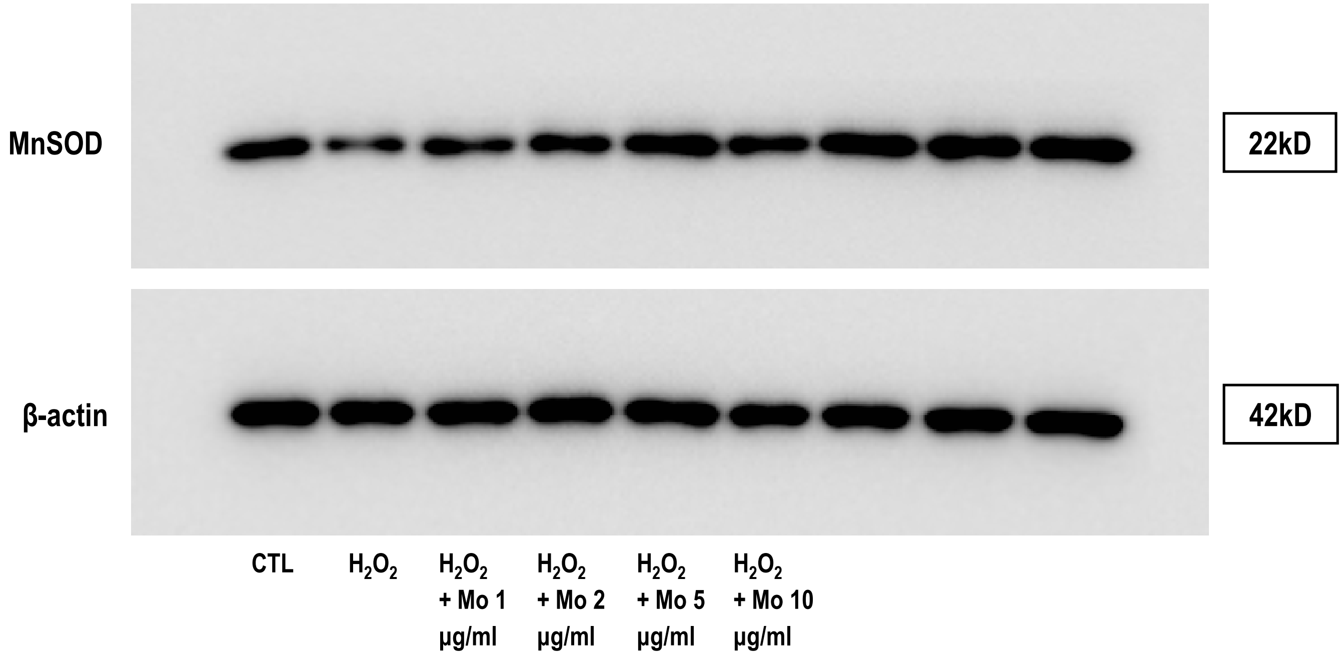

1. 2019 08 29 Western Blot (uncropped)

MnSOD (uncropped)

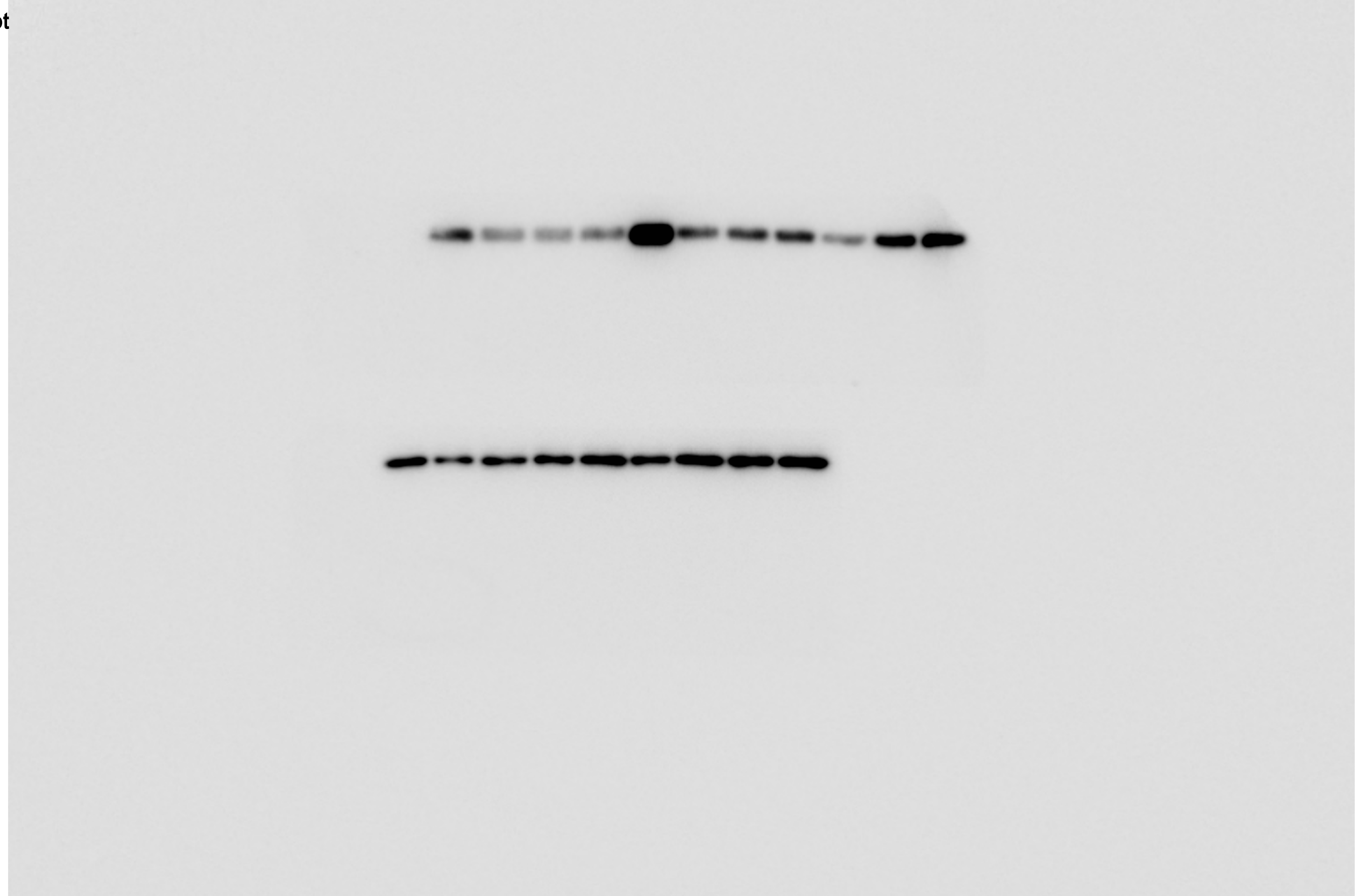

1. 2019 08 29 Western Blot (uncropped)

β-actin (uncropped)

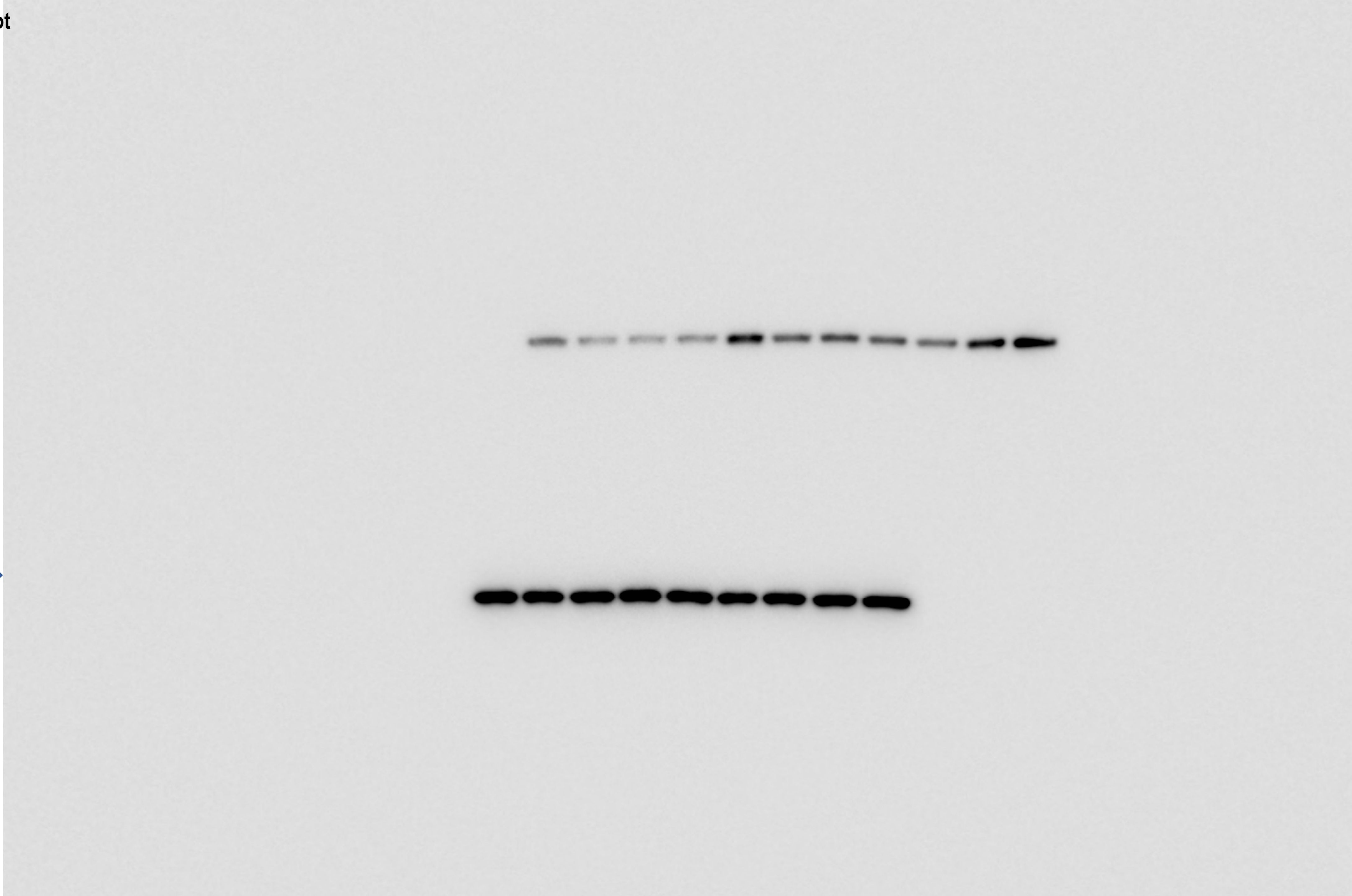

2. 2019 09 11 Western Blot

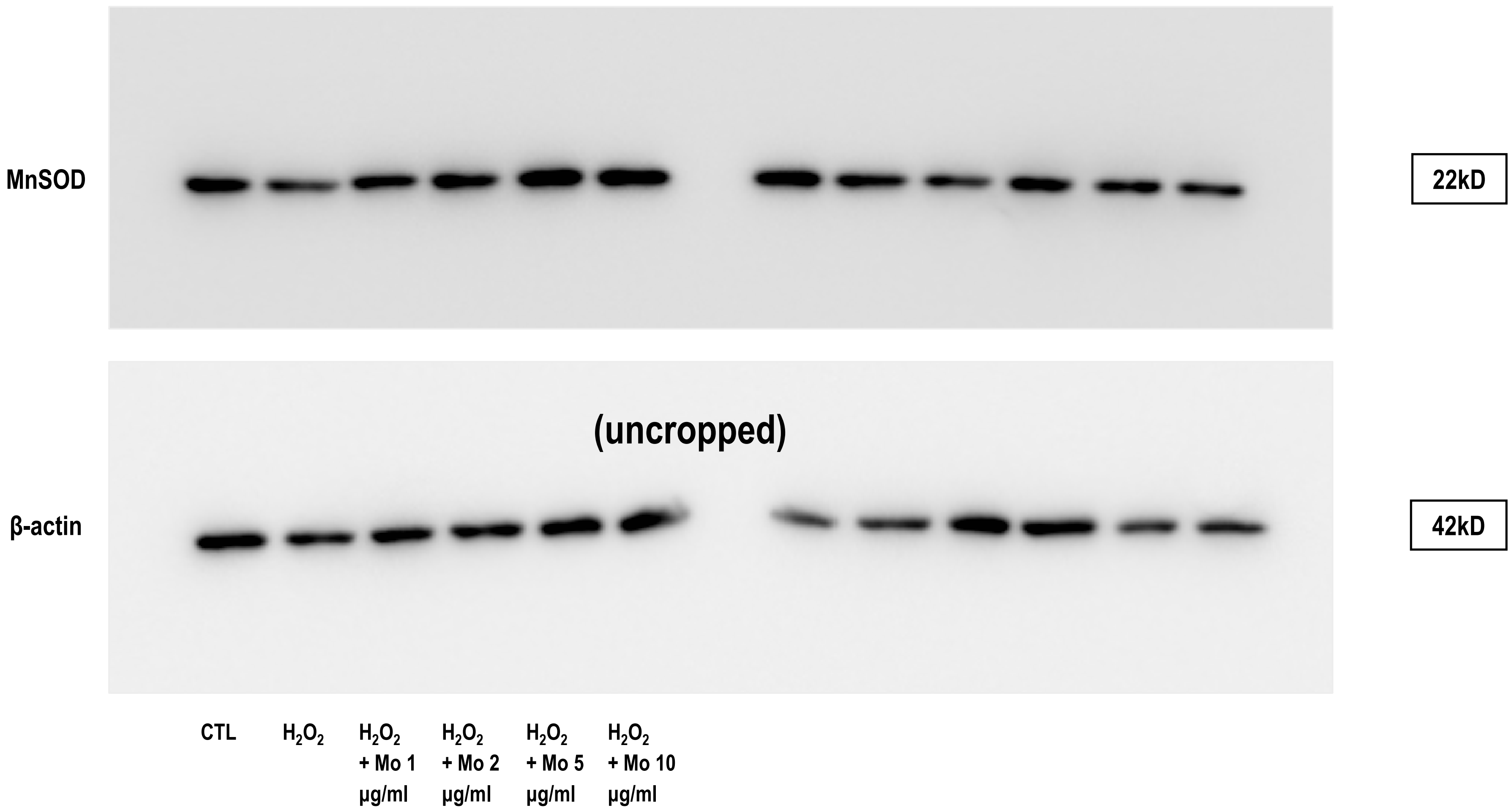

2. 2019 09 11 Western Blot (uncropped)

MnSOD

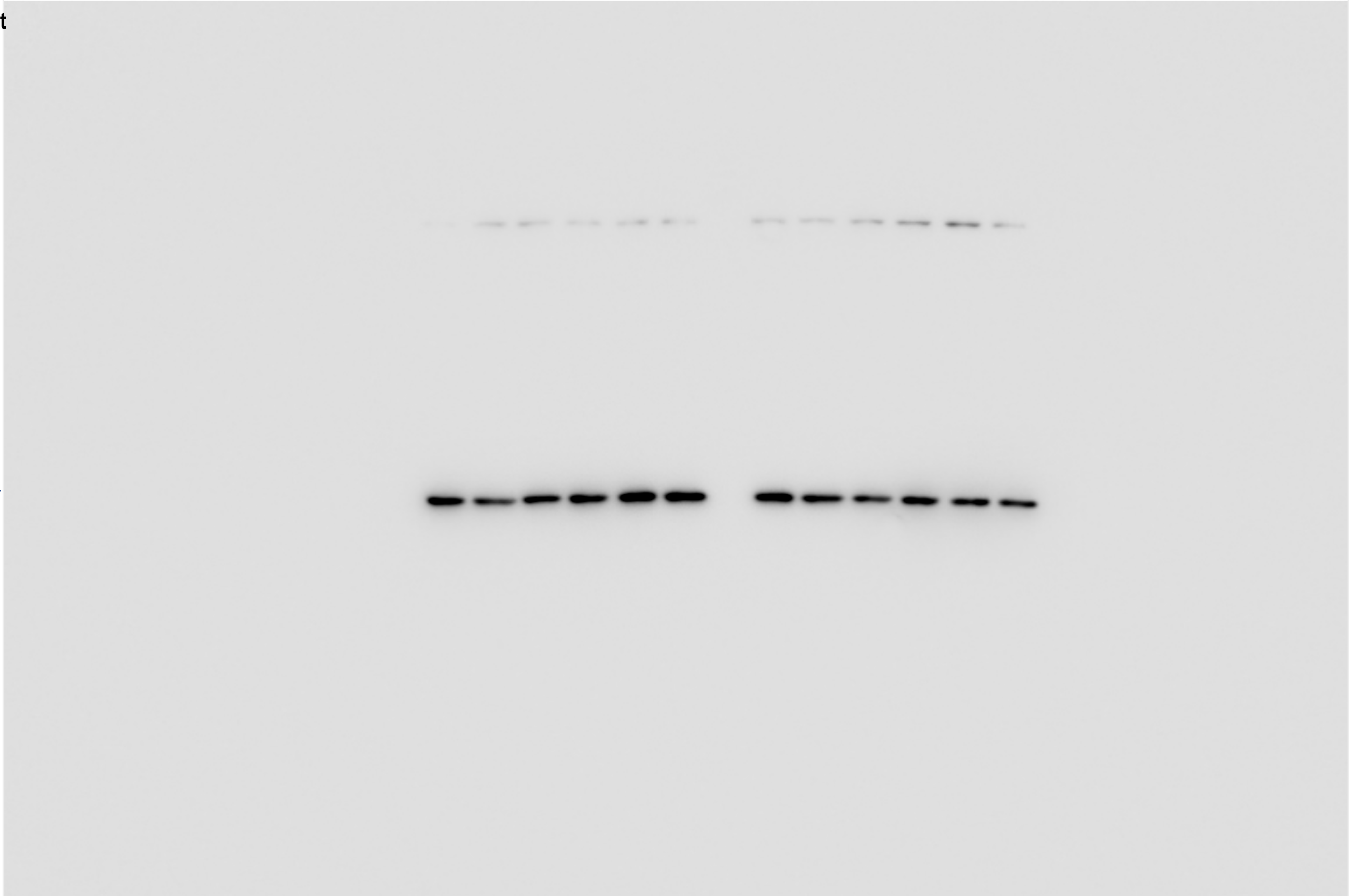

2. 2019 09 11 Western Blot (uncropped)

β-actin

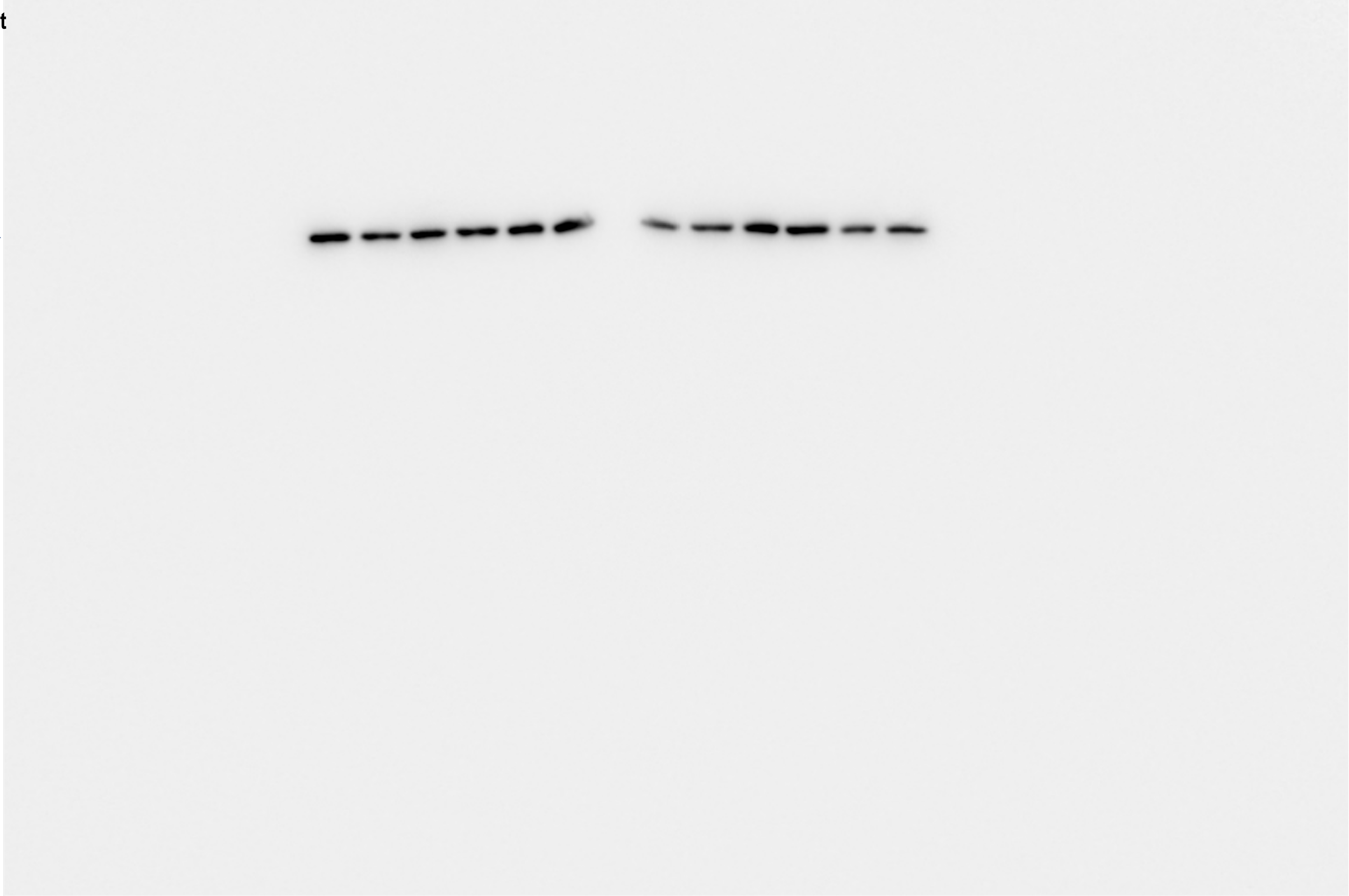

3. 2020 02 23 Western Blot

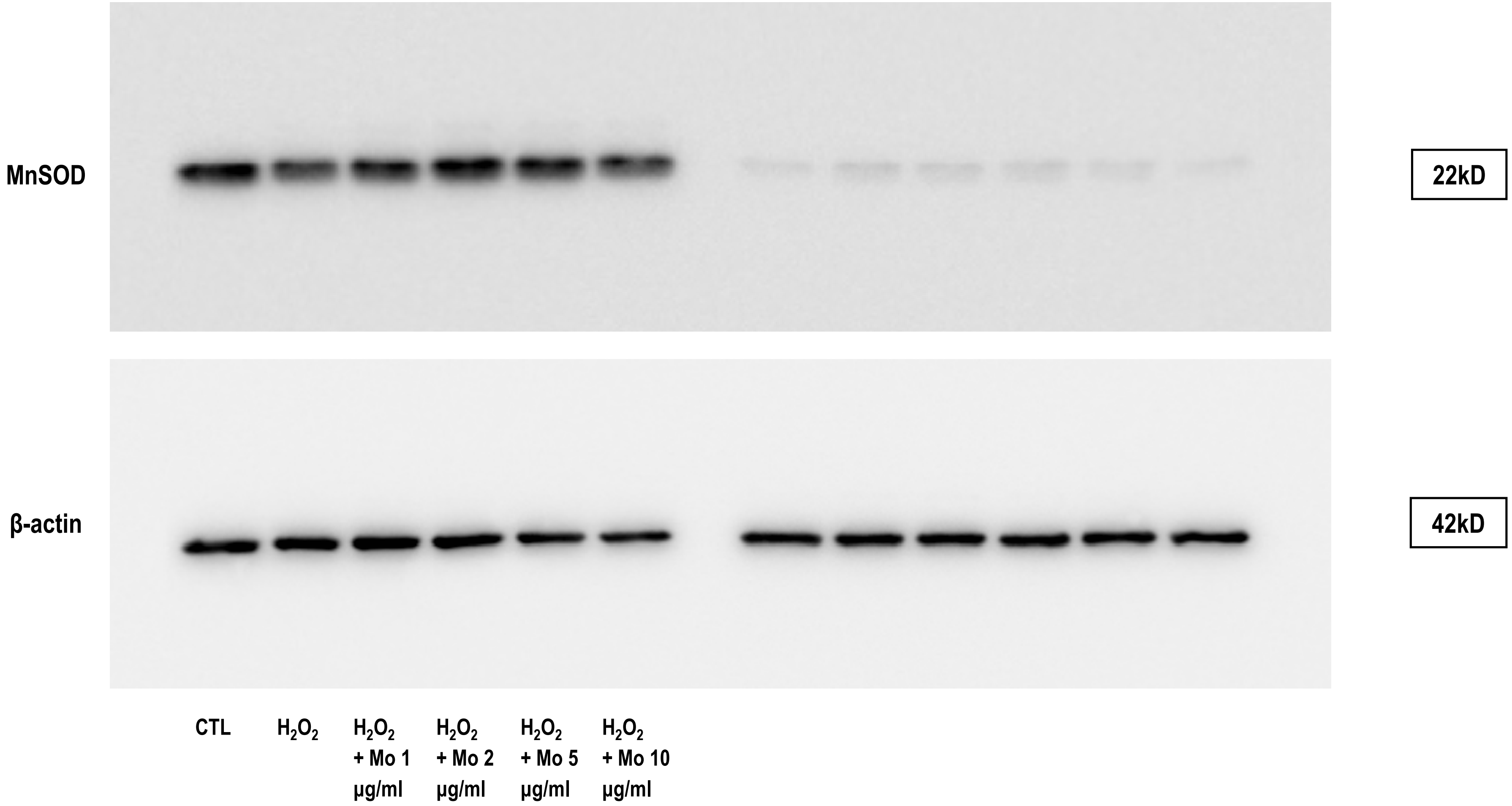

3. 2020 02 23 Western Blot (uncropped)

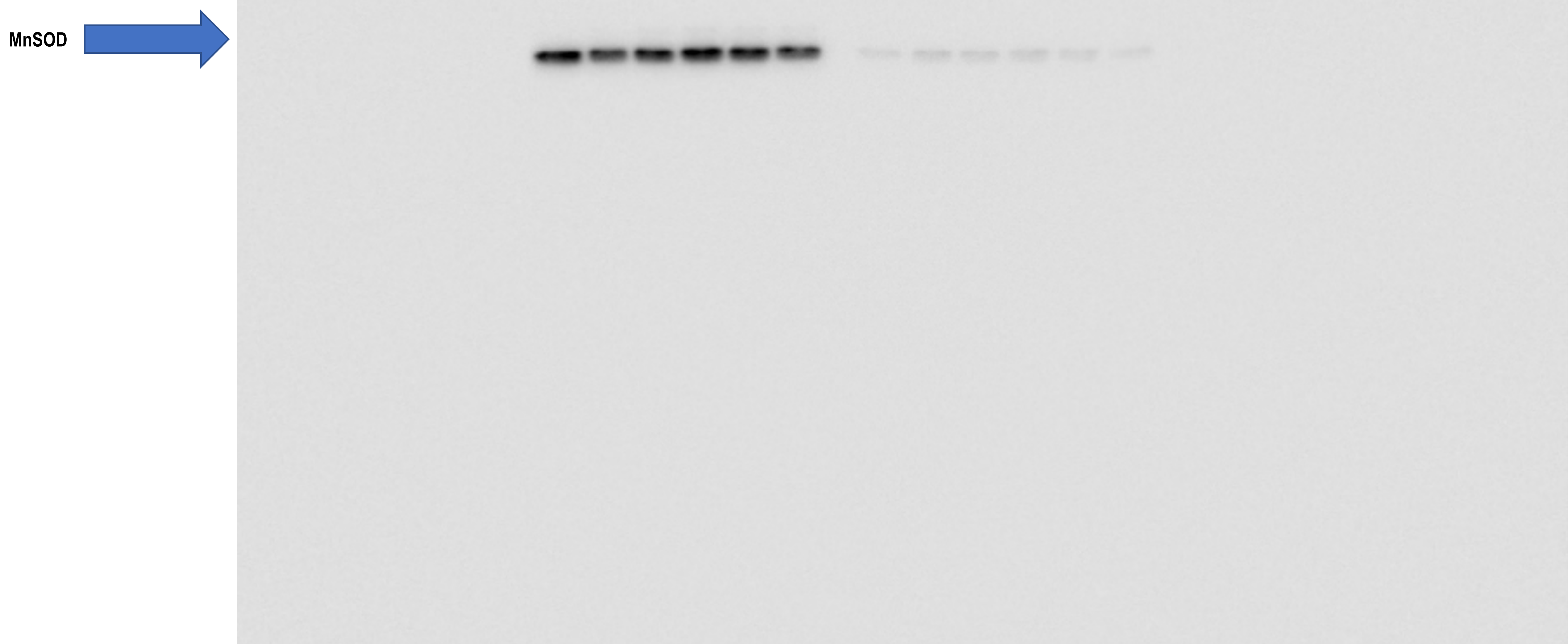

3. 2020 02 23 Western Blot (uncropped)

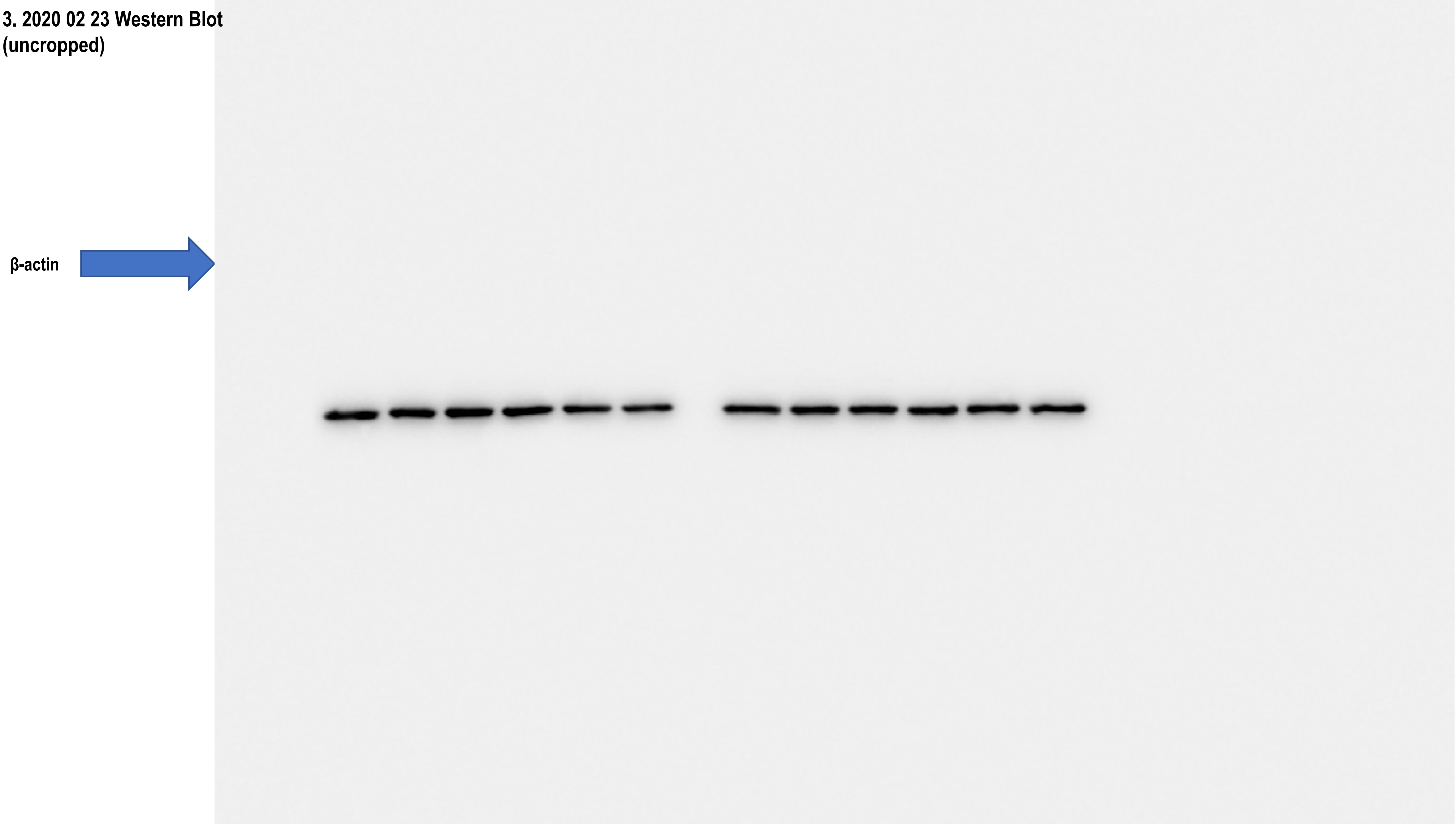

Supplement: S1 Raw images — (PDF) [file pone.0306025.s007.pdf]
