# Supplementary material for: Antioxidative effects of molybdenum and its association with reduced prevalence of hyperuricemia in the adult population
Source: PLoS One. 2024 Aug 1;19(8):e0306025. doi: 10.1371/journal.pone.0306025 (PMC11293656; doi:10.1371/journal.pone.0306025)
Supplement: Attachment — Submitted filename: Review_6.pdf [file pone.0306025.s010.pdf]

Comments on “Antioxidative effects of Molybdenum and its association with reduced prevalence of hyperuricemia in the adult population”

1. Regarding the response to reviewer #1, my comment will be that this manuscript depicts a plausible correlation between urinary excretion rate of molybdenum and disease occurrence of hyperuricemia and gout. However, any strong biological mechanistic aspect of contribution of molybdenum in these above-mentioned diseases manifestation is lacking.

2. In response to the comments from reviewer#2, addition of the mentioned papers showing both discrepancies and similarities, with respect to the findings on the role of Molybdenum levels, intake, etc. in the disease conditions, mentioned in the manuscript is appreciated.

3. For the comments on Reviewer#3, it is agreeable that population size is critically important and impactful in these kinds of studies. So a well-defined population size and vigorous statistical analysis is absolutely critical. Subdivision of the population based on multivariable model is a neat way to show the data, but I would recommend a pie or bar graph or any other graphical representation besides the tables of data provided. Like, a volcano plot should be able to show any correlation or overlap between different subgroups also.

4. For both figure 1 and figure 2a, I would suggest that the graphs be plotted as a Kaplan-Meier surviaval plot because it is a better way of scientific representation of data.

5. For figure 2c, does the bar graph labelling order in the X-axis match with the loading order of the raw data of the Western blot of the protein MnSOD?
