# Supplementary material for: Antioxidative effects of molybdenum and its association with reduced prevalence of hyperuricemia in the adult population
Source: PLoS One. 2024 Aug 1;19(8):e0306025. doi: 10.1371/journal.pone.0306025 (PMC11293656; doi:10.1371/journal.pone.0306025)
Supplement: Attachment — Submitted filename: Rebuttal letter 240410 (Response to Reviewers).docx [file pone.0306025.s011.docx]

**Comments from the Editor and Reviewers**

Thank you for submitting your manuscript to PLOS ONE. After careful consideration, we feel that it has merit but does not fully meet PLOS ONE’s publication criteria as it currently stands. Therefore, we invite you to submit a revised version of the manuscript that addresses the points raised during the review process.

Your manuscript was reviewed by a new reviewer as original reviewer was not available. Few minor comments were provided. Please address all comments as appropriate.

Please submit your revised manuscript by Mar 14 2024 11:59PM. If you will need more time than this to complete your revisions, please reply to this message or contact the journal office at plosone@plos.org. When you're ready to submit your revision, log on to https://www.editorialmanager.com/pone/ and select the 'Submissions Needing Revision' folder to locate your manuscript file.

Please include the following items when submitting your revised manuscript:

A rebuttal letter that responds to each point raised by the academic editor and reviewer(s). You should upload this letter as a separate file labeled 'Response to Reviewers'.

A marked-up copy of your manuscript that highlights changes made to the original version. You should upload this as a separate file labeled 'Revised Manuscript with Track Changes'.

An unmarked version of your revised paper without tracked changes. You should upload this as a separate file labeled 'Manuscript'.

If you would like to make changes to your financial disclosure, please include your updated statement in your cover letter. Guidelines for resubmitting your figure files are available below the reviewer comments at the end of this letter.

If applicable, we recommend that you deposit your laboratory protocols in protocols.io to enhance the reproducibility of your results. Protocols.io assigns your protocol its own identifier (DOI) so that it can be cited independently in the future. For instructions see: https://journals.plos.org/plosone/s/submission-guidelines#loc-laboratory-protocols. Additionally, PLOS ONE offers an option for publishing peer-reviewed Lab Protocol articles, which describe protocols hosted on protocols.io. Read more information on sharing protocols at https://plos.org/protocols?utm_medium=editorial-email&utm_source=authorletters&utm_campaign=protocols.

We look forward to receiving your revised manuscript.

Kind regards,

Partha Mukhopadhyay, Ph.D.

Section Editor

PLOS ONE

Reviewer Comments:

Reviewer #4: Review

4-1. Regarding the response to reviewer #1, my comment will be that this manuscript depicts a plausible correlation between urinary excretion rate of molybdenum and disease occurrence of hyperuricemia and gout. However, any strong biological mechanistic aspect of contribution of molybdenum in these above-mentioned diseases manifestation is lacking.

: Thank you for valuable comment. In this study, we did not show the direct mechanism of the uric acid lowering effects of molybdenum. This is the study's main limitation. However, we found novel findings of a decreased prevalence of hyperuricemia according to the increase in urinary molybdenum levels. We performed thorough statistical analysis, including sensitivity analysis and subgroup analysis, with other metal (antimony and tungsten) controls. In addition, we suggested the antioxidative effect of molybdenum in both the NHANES cohort study (associated with decreased levels of GGT, an index of systemic oxidative stress) and *in vitro* experiments. In the discussion section, we added the limitations and strengths of this study as follows: “Our cross-sectional analysis of the adult population is inherently unable to determine the causal relationship between molybdenum excretion and hyperuricemia.” , “Although we found the anti-oxidative effect of molybdenum both in the cohort study (association with decreased levels of GGT) and in cellular experiments (associated with decreased levels of ROS and increased levels of anti-oxidation enzyme levels), these findings are not direct evidence of the uric acid-lowering effects of molybdenum.” , “Animal experiments or clinical studies to determine molybdenum’s effects on urinary or intestinal uric acid excretion and urate transporters might be helpful.”

4-2. In response to the comments from reviewer#2, addition of the mentioned papers showing both discrepancies and similarities, with respect to the findings on the role of Molybdenum levels, intake, etc. in the disease conditions, mentioned in the manuscript is appreciated.

: Thank you for the valuable comments. The strength of this study is that increased molybdenum intake is associated with lower uric acid levels and a lower frequency of hyperuricemia in the physiologic condition of the general population with normal molybdenum intake. As mentioned in the Introduction and Discussion section, there is a known risk of hyperuricemia or gout at high doses of molybdenum (10 to 15 mg/day) that exceed the usual intake [Recommended Dietary Allowance (RDA) is 45 μg/day or Tolerable Upper Intake Level (UL) is 2,000 μg/day] [11,12]. However, there have also been reports that no increased risk of hyperuricemia or gout has been observed at standard doses [13,14]. In contrast to these previous studies, our results show that increased intake or exposure, expressed as an increase in urinary molybdenum, is associated with a decrease in serum uric acid levels and a decrease in the prevalence of gout.

We suggest that the differences in molybdenum intake may explain why our results differ from other previous studies. During this second revision, we could find additional papers that linked high intakes of molybdenum to increased uric acid and gout. Selde´n et al. reported that long-term occupational exposure to molybdenum increased serum uric acid levels and induced gout-like symptoms. In addition, avoidance of molybdenum exposure improved hyperuricemia and gout (Occup Med (Lond). 2005 Mar;55(2):145-8. doi: 10.1093/occmed/kqi018).

In the Discussion section, we added the related articles and reasonable explanations as follows: “This negative correlation between urinary molybdenum levels and serum uric acid concentration is a novel insight that contradicts previous studies. Several previous studies reported that molybdenum overexposure contributes to the development of hyperuricemia and gout, possibly via xanthine oxidase hyperactivity [10-12]. Nevertheless, our results indicate that the risk of hyperuricemia is reduced according to the increase in urinary molybdenum levels. These discrepancies might be derived from the difference in molybdenum intake amounts. In conditions where large amounts of molybdenum are ingested beyond the physiologic intake, such as in molybdenum intoxication, blood uric acid concentrations are increased by an intake that exceeds the increase in urinary uric acid excretion caused by molybdenum. Hyperuricemia was reported by Koval’skii et al. in patients with molybdenum poisoning; the patients' molybdenum intake was found to be 10 to 15 mg/day [11]. This greatly exceeds the molybdenum RDA of 45 μg/day and the tolerable UL of 2,000 μg/day. Selde´n et al. reported that long-term occupational exposure to molybdenum increased serum uric acid levels and induced gout-like symptoms. In addition, avoidance of molybdenum exposure improved hyperuricemia and gout (Occup Med (Lond). 2005 Mar;55(2):145-8. doi: 10.1093/occmed/kqi018). Deosthale et al. examined the association between molybdenum intake and uric acid levels in four volunteers and found no significant association at an intake amount in the 1.5 mg/day range [14]. Chappell et al. discovered a decrease in blood uric acid levels at a molybdenum intake of 7 μg/kg/day, which is near physiologic intake and is consistent with this study’s findings [38].

4-3. For the comments on Reviewer#3, it is agreeable that population size is critically important and impactful in these kinds of studies. So, a well-defined population size and vigorous statistical analysis is absolutely critical. Subdivision of the population based on multivariable model is a neat way to show the data, but I would recommend a pie or bar graph or any other graphical representation besides the tables of data provided. Like, a volcano plot should be able to show any correlation or overlap between different subgroups also.

: Thank you for the valuable comment. In response to the reviewer's request for rigorous statistical tests during the initial revision, we conducted sensitivity analyses and subgroup analyses based on age category, sex, and BMI category. These additional statistical analyses provided further insight on the substantial connection between urine molybdenum levels and hyperuricemia. In this second revision, we selected forest plots as a more intuitive method to display these findings and provided them as the main Figure 3.

**Figure 3.** Forest plot for the risk of hyperuricemia due to the increased urinary molybdenum levels. The risk of hyperuricemia associated with urinary molybdenum levels (adjusted odds ratio of the 4^th^ quartile vs. 1^st^ quartile of urinary molybdenum) was compared according to age, sex, and BMI. Multivariate logistic regression analysis was adjusted for age, sex, ethnicity, BMI, diabetes mellitus, hypertension, and estimated glomerular filtration rate.

4-4. For both figure 1 and figure 2a, I would suggest that the graphs be plotted as a Kaplan-Meier survival plot because it is a better way of scientific representation of data.

: Thank you for the valuable comment. In general, Kaplan-Meier analysis is suitable for analyzing outcomes with event time. However, this study reports novel findings of a significant association between urinary metal (molybdenum) concentrations and serum uric acid levels from the NHANES cohort study with a cross-sectional design. In the absence of information on the time of hyperuricemia’s onset, we performed logistic regression analysis (instead of Cox regression or Kaplan-Meir analysis) and the results was visualized using the spline curve in Figure 1. In the current dataset, Kaplan-Meier or Cox regression is impractical due to the lack of information on the onset time of hyperuricemia (cross-sectional analysis design).

In Figure 2a, the results of kidney tubular cell viability according to molybdenum concentration are also presented, but there are no differences in the event time (all viability data was measured 24 hours after molybdenum exposure), so we believe that the current presentation method is appropriate.

4-5. For figure 2c, does the bar graph labelling order in the X-axis match with the loading order of the raw data of the Western blot of the protein MnSOD?

: Thank you for the important comment. The loading order of the western blot in Figure 2C (upper panel) matches exactly with the order of the x-axis in the quantification results for the western blot shown below (lower panel). All of the raw data for the western blot is included in the Supplements, and the loading order matches X-axis labeling of Figure 2-C. To clarify the results description, the authors corrected Figure 2-C’s figure legend as follows: The authors corrected Figure 2-C’s figure legend as follows: (C) Western blotting for manganese superoxide dismutase (MnSOD). Representative western blot image (upper panel) and measurement of western blot results (lower panel). The loading order of the western blot (upper panel) matches exactly with the order of the X-axis in the quantification results (lower panel). Molybdenum treatment increased the expression of cellular antioxidative MnSOD (n = 3 per group). Oxidative stress was induced in HK-2 cells with 100 μM H_2_O_2_, and molybdenum was administered at 1, 2, 5, and 10 μg/mL. Bar graph shows the mean and standard deviation levels. ^*^*P* < 0.05, ^**^*P* < 0.01; Mo, molybdenum; ROS, reactive oxygen species

The figures in the supplementary data also include the western band loading sequences.
